# Seamlessly Splicing Metallic Sn*_x_*Mo_1−_ *_x_*S_2_ at MoS_2_ Edge for Enhanced Photoelectrocatalytic Performance in Microreactor

**DOI:** 10.1002/advs.202002172

**Published:** 2020-11-16

**Authors:** Gonglei Shao, Yizhen Lu, Jinhua Hong, Xiong‐Xiong Xue, Jinqiang Huang, Zheyuan Xu, Xiangchao Lu, Yuanyuan Jin, Xiao Liu, Huimin Li, Sheng Hu, Kazu Suenaga, Zheng Han, Ying Jiang, Shisheng Li, Yexin Feng, Anlian Pan, Yung‐Chang Lin, Yang Cao, Song Liu

**Affiliations:** ^1^ Institute of Chemical Biology and Nanomedicine (ICBN) State Key Laboratory of Chemo/Biosensing and Chemometrics College of Chemistry and Chemical Engineering Hunan University Changsha 410082 P. R. China; ^2^ State Key Laboratory of Physical Chemistry of Solid Surfaces Collaborative Innovation Center of Chemistry for Energy Materials (iChEM) College of Chemistry and Chemical Engineering Xiamen University Xiamen 361005 P. R. China; ^3^ Nanomaterials Research Institute National Institute of Advanced Industrial Science and Technology (AIST) Tsukuba 305‐8565 Japan; ^4^ Hunan Provincial Key Laboratory of Low‐Dimensional Structural Physics and Devices School of Physics and Electronics Hunan University Changsha 410082 P. R. China; ^5^ School of Physics and Optoelectronics Xiangtan University Xiangtan 411105 P. R. China; ^6^ Shenyang National Laboratory for Materials Science Institute of Metal Research Chinese Academy of Sciences Shenyang 110016 P. R. China; ^7^ School of Material Science and Engineering University of Science and Technology of China Hefei 230026 P. R. China; ^8^ Key Laboratory for Micro‐Nano Physics and Technology of Hunan Province State Key Laboratory of Chemo/Biosensing and Chemometrics College of Materials Science and Engineering Hunan University Changsha 410082 P. R. China; ^9^ School of Physics and Electronics Hunan University Changsha 410082 P. R. China; ^10^ International Center for Young Scientists (ICYS) National Institute for Materials Science (NIMS) Tsukuba 305‐0044 Japan

**Keywords:** chemical vapor deposition, covalent bonds, heteroatom doping, metal–semiconductor heterostructures, photoelectrocatalytic performance

## Abstract

Accurate design of the 2D metal–semiconductor (M–S) heterostructure via the covalent combination of appropriate metallic and semiconducting materials is urgently needed for fabricating high‐performance nanodevices and enhancing catalytic performance. Hence, the lateral epitaxial growth of M–S Sn*_x_*Mo_1−_ *_x_*S_2_/MoS_2_ heterostructure is precisely prepared with in situ growth of metallic Sn*_x_*Mo_1−_ *_x_*S_2_ by doping Sn atoms at semiconductor MoS_2_ edge via one‐step chemical vapor deposition. The atomically sharp interface of this heterostructure exhibits clearly distinguished performance based on a series of characterizations. The oxygen evolution photoelectrocatalytic performance of the epitaxial M–S heterostructure is 2.5 times higher than that of pure MoS_2_ in microreactor, attributed to the efficient electron–hole separation and rapid charge transfer. This growth method provides a general strategy for fabricating seamless M–S lateral heterostructures by controllable doping heteroatoms. The M–S heterostructures show increased carrier migration rate and eliminated Fermi level pinning effect, contributing to their potential in devices and catalytic system.

## Graphical abstract

The epitaxial growth of metal–semiconductor (M–S) Sn*_x_*Mo_1−_ *_x_*S_2_/MoS_2_ heterostructure is precisely prepared with in situ growth of metallic Sn*_x_*Mo_1−_ *_x_*S_2_ at semiconductor MoS_2_ edge via one‐step chemical vapor deposition method. The oxygen evolution photoelectrocatalytic performance of the epitaxial M–S heterostructure is 2.5 times higher than that of pure MoS_2_ in microreactor, attributed to the efficient electron–hole separation and rapid charge transfer.

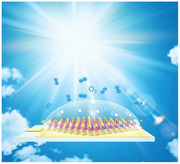

Excessive energy consumption leads to worsening environmental pollution and global warming. As a carrier of recyclable solar energy, sustainable production of hydrogen from water splitting has been intensively investigated.^[^ ^1^, ^2^, ^3^ ^]^ Nanostructured semiconductors have been normally used as photoelectric catalysts for solar hydrogen production, however, they suffered from the inferior characteristics, including unsatisfactory stability and photocorrosion under light illumination.^[^ ^3^, ^4^ ^]^ The total energy conversion efficiency has been still far from ideal situation, in which the photoelectric catalysts should concurrently provide strong absorption range of sunlight, rapid charge transport, high density‐active sites, suitable band gap and band energy levels, large specific surface area, as well as they should have low cost and be environmental friendly.^[^ ^1^, ^2^, ^5^ ^]^

2D transition metal dichalcogenides (TMDs) materials exhibited the quantum‐confined effect in the planar dimension with minimum interlayer interactions.^[^ ^6^, ^7^ ^]^ Their ultrathin structure with large surface area provided great charge migration rate, strong optical absorption range with tunable electronic structure, and high mechanical flexibility.^[^ ^8^ ^]^ Interlayer stacking or intralayer splicing heterostructures have been demonstrated as effective strategy to precisely tailor the electronic properties.^[^ ^9^ ^]^ Thus, their photoelectrocatalytic performance for water splitting can be availably improved.^[^ ^2^, ^10^, ^11^ ^]^ For example, 2D semiconducting molybdenum disulfide (MoS_2_) was a favorable candidate with appropriate energy band gap (1.82 eV in monolayer) for solar absorption.^[^ ^4^, ^12^, ^13^ ^]^ More importantly, 2D MoS_2_ with high light transparency possessed outstanding electronic features, manifested as high carrier mobility and tunable charge‐carrier behavior.^[^ ^12^, ^14^ ^]^ However, there are still several obstacles waiting to be addressed, such as photocorrosion, poor stability, and low photon‐conversion efficiency in visible light range, which have greatly limited their potential applications.^[^ ^4^, ^15^ ^]^ These problems have been mainly due to their inefficient photogenerated electron–hole separation and low transportation efficiency, originating from the higher electron transfer barrier at the interface between the semiconductors and the electrodes.^[^ ^16^ ^]^ Hence, it is of great importance to optimize the interface structure to realize the fast electron–hole separation, minimize the photogenerated electron–hole recombination, and prolong the life of electron or hole for improving photoelectrocatalytic performance.

The MoS_2_‐based lateral heterointerfaces have been found to markedly enhance H_2_ or O_2_ production efficiency.^[^ ^13^, ^17^, ^18^ ^]^ A more elaborate design was the epitaxial M–S heterostructures, which were considered as intriguing building blocks for low‐power, high‐performance, and flexible electronic and optoelectronic devices.^[^ ^19^, ^20^, ^21^ ^]^ In the artificially in‐plane M–S heterostructures, the metallic 2D materials governed internal electrical transport, and the covalent connection replaced traditional physical electrode contact, which can dramatically improve charge injection, thus enhancing electrical performance.^[^ ^22^ ^]^ The covalently bonded contacts significantly lowered the Schottky barrier height, achieving ohmic characteristics by eliminating the Fermi level pinning effect.^[^ ^23^, ^24^ ^]^ Furthermore, seamless stitching with metallic materials as contact electrodes can change the charge transfer kinetics and facilitate rapid electronic transmission across the interface.^[^ ^25^ ^]^ Hence, the direct in situ synthesis of seamless splicing 2D epitaxial M–S lateral heterostructure through covalent bond was highly desirable for effectively reducing the contact resistance and improving the carrier migration rate at interface to promote the photogenerated electron–hole separation in photoelectric catalytic system.

Chemical vapor deposition (CVD) is a powerful approach for preparing lateral epitaxial 2D heterostructures and stacking superlattices. However, the preparation of laterally epitaxial M–S heterostructures with atomically sharp interface through one‐pot or multi‐step methods has been still limited by the lattice mismatch and atomic compatibility.^[^ ^21^, ^25^, ^26^ ^]^ In this work, we developed a heteroatom Sn doping engineering to prepare monolayer Sn*_x_*Mo_1−_ *_x_*S_2_/MoS_2_ M–S epitaxial heterostructure via one‐pot CVD growth. In this method, seamlessly spliced metallic Sn*_x_*Mo_1−_ *_x_*S_2_ was constructed at MoS_2_ edge by covalent bonds. A measurement system was developed in a homemade microreactor to explore the working mechanism of this 2D crystal in oxygen evolution reaction (OER)‐based photoelectrocatalysis. The M–S heterostructure crystal flakes were first transferred to Ti electrode on quartz glass sheets and then connected in the microreactor. The microreactor has following advantages: 1) A single tiny sample can be tested, and the specifically designed structure can be investigated and quantified.^[^ ^27^ ^]^ 2) Electron–hole instantaneous recombination resulted from mutual interference and hybridization materials can be effectively avoided in microreactor, thus improving light utilization rate per unit area. 3) The catalytic mechanism and charge transfer can be easily monitored in micromodel reaction for building the structure–activity relationship.^[^ ^2^, ^28^, ^29^ ^]^ This epitaxial M–S Sn*_x_*Mo_1−_ *_x_*S_2_/MoS_2_ heterostructure showed an improved OER photoelectrocatalytic performance of 2.5 times, resulted from the efficient electron hole separation and rapid electron transfer with low resistance at the M–S interface. The in‐plane seamlessly splicing growth with 2D semiconductors and metallic alloys by covalent bonding provided a general strategy for the preparation of M–S epitaxial heterostructures. This artificial structure will be conducive to improving photoelectrocatalytic performance, accompanied by increased carrier migration rate.

It has been reported that heteroatom doping can induce the transition of electrical transport behavior of 2D materials, from semiconducting to metallic for M–S heterostructure construction.^[^ ^16^, ^30^ ^]^ The electronic densities of states (DOS) of MoS_2_ and Sn*_x_*Mo_1−_ *_x_*S_2_ calculated with the density functional theory (DFT) method are shown in **Figure** **1**. The projected DOS and electronic densities at the Fermi level for Sn*_x_*Mo_1−_ *_x_*S_2_ confirmed that a slight Sn doping (≈2%) in MoS_2_ could cause a transition from typical semiconductor to metal. This result became the fundamental basis for the development of seamless splicing M–S Sn*_x_*Mo_1−_ *_x_*S_2_/MoS_2_ heterostructures with in‐plane covalent bonds, which can be applied as high‐performance photoelectrocatalyst.^[^ ^16^ ^]^ The semiconductor and metallic alloy have the same phase structure and similar lattice constant, providing an efficient and ingenious design for seamless splicing M–S epitaxial heterostructure.

**Figure 1:**
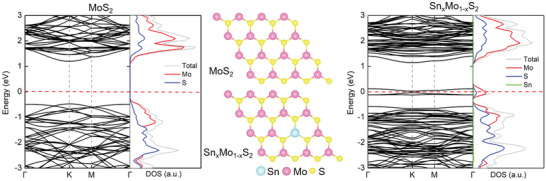
Electronic band structures along the symmetry directions of the Brillouin zone and partial density of states for MoS_2_ and Sn*_x_*Mo_1−_ *_x_*S_2_. (*N*_Sn_/*N*_Mo_ = 1/48). The Fermi levels are labeled with red dashed lines. Image in the middle shows atomic structure schematic diagrams of MoS_2_ and Sn*_x_*Mo_1−_ *_x_*S_2_ used for simulation.

The 2D Sn*_x_*Mo_1−_ *_x_*S_2_/MoS_2_ M–S epitaxial heterostructure was achieved via a facile CVD as shown in **Figure** **2**a. In the meantime, MoS_2_ and Sn*_x_*Mo_1−_ *_x_*S_2_ alloy deposited on SiO_2_/Si substrate separately was also prepared as a comparison (the growth details are provided in Figure S2, Supporting Information). In a typical growth of Sn*_x_*Mo_1−_ *_x_*S_2_/MoS_2_ heterostructure, SnO_2_ and MoO_3_ powders were selected as precursors, and NaCl was also mixed with precursors as catalyst to lower the growth temperature. The two precursors were spaced with an interval of 0.5 cm in the crucible, which prevented the volatile precursor from evaporating and depositing together on the SiO_2_/Si substrate, resulting in the growth of Sn*_x_*Mo_1−_ *_x_*S_2_ alloy. As for the separate precursor powder, the upstream MoO_3_ reaches saturation vapor pressure first, the downstream volatile SnO_2_ reaches saturation vapor pressure later. The two kinds of powders are separated at a certain space, the growth sequences are separated in space and time. Thus, such spatial spacing results in that MoS_2_ are already preferred to grow in the central encounter. While SnS_2_ diffuses and is mixed with MoS_2_, this lead to Sn*_x_*Mo_1−_ *_x_*S_2_ grow that the edge of MoS_2_ for Sn*_x_*Mo_1−_ *_x_*S_2_/MoS_2_ heterostructure.

**Figure 2:**
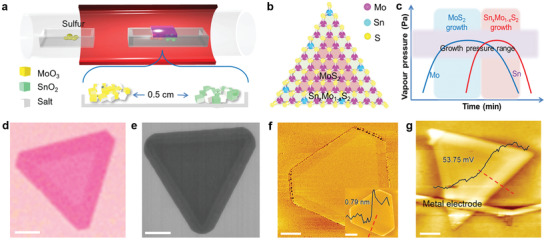
Controllable growth of epitaxial Sn*_x_*Mo_1−_ *_x_*S_2_/MoS_2_M–S heterostructure. a) Growth schematic of Sn*_x_*Mo_1−_ *_x_*S_2_/MoS_2_heterostructure. b) Atomic structure diagram of the lateral heterostructure. Inside: MoS_2_. Outside: Sn*_x_*Mo_1−_ *_x_*S_2_. c) Growth process prediction of the heterostructure. d) Optical image and e) SEM image of Sn*_x_*Mo_1−_ *_x_*S_2_/MoS_2_heterostructures. f) The phase image of AFM. Inset: AFM height image of monolayer heterostructure and corresponding height profile line along the red dashed line. g) KPFM image of the heterostructure with gold electrode. Inset: line profile perpendicular to the interface as marked on the KPFM image with the red dotted line. All the scale bar: 5 µm.

The atomic structure diagram of this Sn*_x_*Mo_1−_ *_x_*S_2_/MoS_2_ heterostructure is displayed in Figure 2b, with the inside core of semiconductor MoS_2_ and outside of Sn‐doped metallic Sn*_x_*Mo_1−_ *_x_*S_2_ alloy. The color difference of atoms clearly distinguished the heterogeneous lateral interface of the structure. After growth, the flakes formed a concentric heterogeneous structure with two different color contrast (Figure 2d; Figure S3, Supporting Information). It can be observed that the color and structure of the heterostructures were obviously different from that of MoS_2_ and Sn*_x_*Mo_1−_ *_x_*S_2_ alloy (Figure S5, Supporting Information). Raman and photoluminescence (PL) spectra were conducted to analyze MoS_2_ and metallic Sn*_x_*Mo_1−_ *_x_*S_2_ (Figures S6 and S8, Supporting Information). After Sn doping, new vibration peaks at 182.9, 220.4, and 346.1 cm^‐1^ were observed in Raman spectra for Sn*_x_*Mo_1−_ *_x_*S_2_. The absence of PL excitation for Sn*_x_*Mo_1−_ *_x_*S_2_ flake indicated their metallic behavior. Besides, in *I*_DS_–*V*_G_ test, the *I*_DS_ was linearly increased with *V*_DS_, independent with *V*_G_, which further demonstrated the metallic feature of Sn*_x_*Mo_1−_ *_x_*S_2_ alloy (Figures S9 and S10, Supporting Information). The results confirmed that small amount of Sn doping caused the transition of MoS_2_ from n‐type semiconductor to metallic Sn*_x_*Mo_1−_ *_x_*S_2_.

The lateral M–S heterostructures were created along the growth sequence indicated in Figure 2c, which were determined by the growth vapor pressure difference of MoS_2_ and SnS_2_ at the same temperature. MoS_2_ reached the saturated vapor pressure first because of the faster volatilization rate and then formed pure MoS_2_ core on the substrate. After that, the vapor pressure of SnS_2_ precursor existed and mixed with Mo to deposit Sn*_x_*Mo_1−_ *_x_*S_2_ on MoS_2_ edges. The M–S heterostructures were formed by the resulting volatility variation and atomic compatibility. The growth mechanism can be clearly confirmed by the thermogravimetric analysis of SnS_2_ and MoS_2_ (Figure S11, Supporting Information), in which Mo source was volatilized preferentially to Sn, and metal component of the vapor above the substrate was Mo and Sn atoms. Meanwhile, the edge of 2D monolayer MoS_2_ has many active suspension S–Mo bonds during growth, which are very conducive to further growth of Sn*_x_*Mo_1−_ *_x_*S_2_ at edge of MoS_2_. Furthermore, Sn*_x_*Mo_1−_ *_x_*S_2_ and MoS_2_ exist almost in the same lattice, this similar lattice can continue to in‐plane growth with no energy barriers and difficulties. Hence, we emphasized that this process should avoid external action during the growth of lateral M–S heterostructures, and maintain ideal conditions for 2H phase epitaxy by doping heteroatoms with minimal complication.^[^ ^31^ ^]^

All the grown lateral M–S heterostructure crystals showed color contrast in core and edge regions with clear interface under optical microscope as shown in Figure 2d. The width of the outer region was similar for crystals in a given growth condition (Figures S3 and S5c, Supporting Information). The interface of heterostructure became much clear under scanning electron microscope (SEM) as shown in Figure 2e (Figure S4, Supporting Information). The secondary electron image indicated the variations of work function and composition between the core and outer ring.^[^ ^32^ ^]^ Under atomic force microscope (AFM), the epitaxial heterostructure exhibited uniform height at ≈0.79 nm monolayer with seamless connection (inset of Figure 2f). Moreover, the corresponding phase image showed clear color contrast in Figure 2f, indicating different elemental composition and structure in core and edge regions. Kelvin probe force microscope (KPFM) was completed to quantitatively analyze the surface potential between core and edge regions of this heterostructure (Figure 2g; Figure S13, Supporting Information). A clear surface potential interface was observed, indicating different work functions between semiconducting MoS_2_ and metallic Sn*_x_*Mo_1−_ *_x_*S_2_. Across the interface junction, the Fermi‐level difference was calculated to be ≈53.7 mV (inset of Figure 2g), implying that the work function of metallic Sn*_x_*Mo_1−_ *_x_*S_2_ was distinctly smaller than that of MoS_2_ relative to the vacuum level. The difference data were basically close to the theoretical calculation for Fermi level and work function of MoS_2_ and Sn*_x_*Mo_1−_ *_x_*S_2_ by DFT (Figure S1 and Table S1, Supporting Information), which was also consistent with the intrinsic characteristics of Fermi level in M–S heterostructures.^[^ ^20^, ^33^ ^]^ Meanwhile, the stability test of this heterostructure was performed in air for 2 weeks (Figure S14, Supporting Information) and Sn*_x_*Mo_1−_ *_x_*S_2_/MoS_2_ flake was further proved to be a lateral epitaxial splicing heterogeneous structure.

The effects of Sn doping for the epitaxial heterostructure were further analyzed and quantified with Raman and PL. In Raman scanning image with 532 nm laser, typical MoS_2_ vibration peaks (E^1^_2g_ peak at 385.4 cm and the A_1g_ at 405.9 cm) were observed in the core region from **Figure** **3**a. While in the edge, besides of MoS_2_ Raman peaks, three new vibration peaks (J_1_, J_2_, and J_3_) appeared at 182.9, 220.4, and 346.1 cm^‐1^, which were associated with the lattice disorder caused by Sn doping for Sn*_x_*Mo_1−_ *_x_*S_2_.^[^ ^34^, ^35^ ^]^ It showed that the Raman intensity ratio between LA(M) at J_2_ and E^1^_2g_ or A_1g_ was inversely proportional to average interdefect/doping distance (*L*_D_).^[^ ^36^, ^37^ ^]^ From the Raman spectra, the edge Sn*_x_*Mo_1−_ *_x_*S_2_ showed a Raman intensity ratio of 0.237 for I(LA)/I(A_1g_) and 0.432 for I(LA)/I(E^1^_2g_), indicating that the Sn doping concentration was at 2.8–4.0%. The crystal showed uniform MoS_2_ at A_1g_ intensity in the whole regions (Figure 3b). Simultaneously, PL spectra at different regions were also collected (Figure 3c). The core showed that a strong PL intensity peak of MoS_2_ appeared at 1.82 eV, without peak at the edge. PL mapping in Figure 3d clearly outlined the interface of core MoS_2_ and PL quenching edge region. By comparing all the above results, it can be inferred that the core was MoS_2_, while the edge was Sn*_x_*Mo_1−_ *_x_*S_2_.

**Figure 3:**
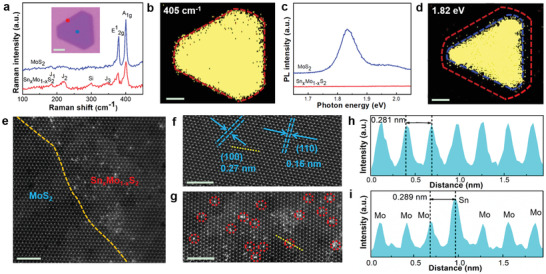
Identification of epitaxial Sn*_x_*Mo_1−_ *_x_*S_2_/MoS_2_ heterostructures. a) Raman spectra and b) Raman mapping of the heterostructures. Inset of (a): optical image of the heterostructure marked by different color dots with signal acquisition area for Raman and PL spectra. Scale bar: 5 µm. c) PL spectra and d) PL mapping of the heterostructures. e) Atomic resolution STEM image taken from the epitaxial M–S heterostructure at the interface. The yellow dotted line indicates the atomic interface. STEM image of f) MoS_2_ at core and g) Sn*_x_*Mo_1−_ *_x_*S_2_ at edge. Scale bar of all STEM images: 2 nm. h) Intensity profile of MoS_2_ from (f) along the yellow dotted lines. i) Intensity profile of Sn*_x_*Mo_1−_ *_x_*S_2_ from (g) along the yellow dotted lines.

For this heterostructure, the structure difference at atomic level between core and edge regions was further explored by high‐angle annular dark‐field scanning transmission electron microscopy (HAADF‐STEM). In the Figure 3e, a perfect atomic interface marked by yellow dotted line was clearly observed. The core region in Figure 3f exhibited hexagonal arrangement with estimated lattice spacing values of 0.16 and 0.27 nm, corresponding to the (110) and (100) plane of MoS_2_, respectively, which were consistent with previous reports.^[^ ^34^, ^38^ ^]^ In edge region, the Sn (*N* = 50, red dotted circles in Figure 3g) atoms can be easily distinguished according to the STEM contrast. It was observed that Sn dopants with bright spots were homogeneously dispersed in MoS_2_ lattice to form Sn*_x_*Mo_1−_ *_x_*S_2_ alloy. The intensity of the atomic brightness from the cross section also confirmed the structure difference (Figure 3h,i). After doping with Sn, the adjacent Mo‐Mo distance of 0.281 nm was increased to Mo–Sn 0.289 nm, resulted in lattice expanding. Based on the statistical analysis, we found that the doping concentration of Sn was about 3.6%, which was very close to data of Raman prediction and DFT theoretical calculation. X‐ray photoelectron spectroscopy (XPS) characterization further confirmed the existence of S–Sn bond existing (Figure S15, Supporting Information).

After the seamlessly spliced M–S Sn*_x_*Mo_1−_ *_x_*S_2_/MoS_2_ structure was proved, the photoelectrocatalytic properties of the heterostructure was next investigated by evaluating its OER performance (4OH^−^ + 4h^+^ = 2H_2_O + O_2_). To that end, we prepared devices with the schematics as depicted in **Figure** **4**a. The 2D heterostructures were transferred to quartz substrates and worked as photoanodes (Figure S16, Supporting Information). Linear‐sweep voltammetry characteristics showed a photocurrent on‐off ratio of 4, with an onset potential *V*_on_ of 0.15 V and a current density *J*_on_ > 0.8 mA cm^−2^ (at 1.23 V vs RHE). These values suggested the heterostructure a significantly better photoanode than MoS_2_ nanosheets (with measured *V*_on_ of 0.22 V and *J*_on_ of 0.2 mA cm^−2^, in agreement with previous report).^[^ ^39^, ^40^ ^]^ In addition, the high *J*_on_ had a rapid response to visible light illumination with a response time <100 ms. With long time illumination up to 2 h, no detectable *J*_on_ decay (within our measurement error of 5%) was observed. The increase of *J*_on_ in the first 0.2 h may be attributed to the thermal effect of light illumination that led to an increased anode temperature.^[^ ^41^ ^]^ In contrast, MoS_2_ anode demonstrated a photocurrent decay by 70%, in agreement with the reported photocorrosion‐induced poor long‐term stability of 2D MoS_2_ crystals.^[^ ^39^, ^42^ ^]^ As a control experiment, the OER performance of Sn*_x_*Mo_1−_ *_x_*S_2_ was also evaluated. Despite of the conductive nature of the Sn*_x_*Mo_1−_ *_x_*S_2_ nanosheet, the low *J*_on_ and high *V*_on_ indicated that the doped Sn atoms made negligible effects on OER reactions.

**Figure 4:**
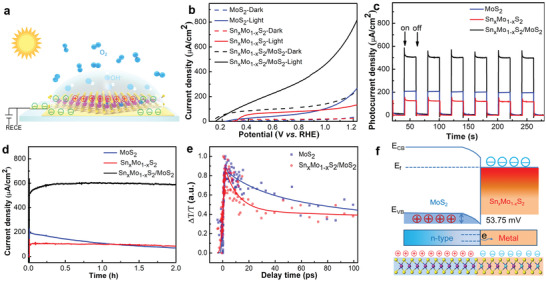
Photoelectrocatalytic performance of Sn*_x_*Mo_1−_ *_x_*S_2_/MoS_2_ heterostructure as a photoanode. a) Schematic of the photoelectrocatalysis measurements. The Sn*_x_*Mo_1−_ *_x_*S_2_/MoS_2_‐on‐quartz sample was connected to the external circuit with Ti electrodes. All the measurements were performed in 0.5mNa_2_SO_4_ solutions. The light illumination was produced using a 300 W Xe lamp unless otherwise specified. b) Linear‐sweep voltammogram curves of different anodes. Solid line: light irradiation. Dashed line: dark. c) Current density as a function of time measured at 1.23 V versus RHE. “On” and “off” marked in the figure represent illumination on and off, respectively. d) Chronoamperometry measurements at 1.23 V versus RHE with laser power density of 100 mW cm^−2^. e) Normalized TAS intensity measured as a function of time under illumination of 400 nm laser with power of 0.35 µW. The pump pulse was set at 3.1 eV, tuned slightly above the band gap of MoS_2_.^[^ ^44^, ^45^ ^]^ f) Schematic of the band profile for Sn*_x_*Mo_1−_ *_x_*S_2_/MoS_2_ heterostructure according to KPFM characterization. Bottom: schematic of the heterostructure's cross section. Purple, cyan, and yellow balls represent Mo, Sn, and S atoms, respectively. Red circles with plus symbol and blue circles with minus symbol represent holes and electrons, respectively.

Our results in Figure 4b–d suggested that the M–S Sn*_x_*Mo_1−_ *_x_*S_2_/MoS_2_ heterostructure was a stable and photocatalytic anode compare to its two individual counterparts. To find out the mechanism, the time‐resolved transient absorption spectra (TAS) were applied to reveal the nature of the photocarrier dynamics of this heterostructure (Figure S19, Supporting Information). As shown in Figure 4e, the decay time constant *τ*_0_ can be obtained by fitting the measured photocarrier dynamics using a single‐exponential function: ΔOD = *c* + *A*_0_ exp (–*t*/*τ*_0_), where ΔOD is the optical intensity, *t* is the time, and *c* and *A*_0_ are the fitting constants. Sn*_x_*Mo_1−_ *_x_*S_2_/MoS_2_ heterostructure exhibited a *τ*_0_ of 10 ± 5 ps, faster than that of MoS_2_ 28 ± 5 ps. Since *τ*_0_ was a direct measurement of the lifetime of photo generated carriers, it indicated an efficient and fast carrier relaxation process in the heterostructure from Figure 4e.

We attributed such carrier behavior to the band structure of the heterostructure, which introduced an additional carrier decay channel for MoS_2_ in the heterostructure (Figure 4f). We recalled that Fermi energy of Sn*_x_*Mo_1−_ *_x_*S_2_ was higher compared to that of MoS_2_ measured from KPFM experiments (Figure 2g). When the two counterparts were in contact, an internal electric field was built at the contact interface where electrons were transferred to the metallic Sn*_x_*Mo_1−_ *_x_*S_2_.^[^ ^43^ ^]^ Under photoillumination, the ohmic contact with low resistance between Sn*_x_*Mo_1−_ *_x_*S_2_ and MoS_2_ further facilitated the fast electron transfer to the external circuits, leaving a hole‐rich 2D anode for OER reaction. This explanation was further supported by our electrochemical impedance spectroscopy (EIS) measurements, in which the interface reaction dynamics of electrode was evaluated (Figure S17, Supporting Information). The charge‐transfer resistance (*R*_ct_) values at the photoanode/electrolyte interface were summarized (Table S2, Supporting Information). Due to the presence of metallic Sn*_x_*Mo_1−_ *_x_*S_2_ with low interface transport resistance, *R*_ct_ of Sn*_x_*Mo_1−_ *_x_*S_2_/MoS_2_ (≈17 Ω) was six orders of magnitude lower compared to that of MoS_2_. Based on above results, the improvement of the overall photoelectrocatalytic water splitting efficiency originated from 1) rapid electron transfer and 2) effective electron–hole separation, both of which benefited from M–S epitaxial heterostructures.

In summary, a slight amount of heteroatom doping in 2D semiconductor can cause a transition from semiconductor to metal in electrical behavior. The epitaxial M–S Sn*_x_*Mo_1−_ *_x_*S_2_/MoS_2_ heterostructures were accurately designed and prepared by sequential growth of monolayer Sn‐doping metallic Sn*_x_*Mo_1−_ *_x_*S_2_ and semiconductor MoS_2_. The epitaxial growth of the metallic Sn*_x_*Mo_1−_ *_x_*S_2_ at MoS_2_ interface can effectively eliminate the Fermi level pinning effect and minimize contact resistance. This M–S Sn*_x_*Mo_1−_ *_x_*S_2_/MoS_2_ heterostructure is conductive to improving the photoelectrocatalytic performance compared to that of MoS_2_. The reason may be the significantly promoted separation of electron holes and effectively increased charge transfer. The accurate doping engineering method provided a general strategy for in situ growth of 2D metallic materials covalent bonding with appropriate 2D semiconductors for self‐assembling M–S epitaxial heterostructures. The seamless splicing of 2D metallic materials at the interface can have great application potentials in high‐performance devices by improving electron injection across the junction. They can also facilitate the applications in catalysis field for enhancing catalytic performance by rapid charge transfer with low‐contact resistances.

## Experimental Section

##### The Growth of M–S Sn*_x_*Mo_1−_ *_x_*S_2_/MoS_2_ Epitaxial Heterostructures

The M–S Sn*_x_*Mo_1−_ *_x_*S_2_/MoS_2_ epitaxial heterostructures were grown with a point‐to‐face metal‐source supply method by a simple atmospheric pressure CVD method. The SiO_2_/Si (270 nm SiO_2_) substrates were cleaned with piranha solution, isopropanol, and DI water, respectively. MoO_3_ (15 mg, ≥99.5%, Sigma‐Aldrich) and NaCl (3 mg, 99.5%, Sinopharm Chemical Reagent Co., Ltd.) were mixed and placed in front of the aluminum trioxide crucible, while SnO_2_ (28 mg, ≥99.0%, Damas‐Beta) and NaCl (5 mg) were uniformly mixed and put behind the MoO_3_/NaCl mixed powder. The two growth powders were placed in the same crucible with space interval of 0.5 cm. The SiO_2_/Si substrate (size: 1 × 2 cm) was located above the powder on the crucible, which was placed in the middle of the furnace. Another crucible with sulfur powder (320 mg, ≥99.5%, Sigma‐Aldrich) was put in upstream, ≈15 cm away from the middle of the furnace. The system was first ventilated with 100 sccm Ar for 10 min to remove other gases in the tube, and maintained an atmosphere with 100 sccm Ar. Then, the furnace was heated up to 695–710 °C for 40 min and maintained for 5 min, and then cooled to room temperature naturally.

##### The Growth of Monolayer MoS_2_ and Sn*_x_*Mo_1−_ *_x_*S_2_ Alloy

The growth methods were basically the same as above. For the growth of MoS_2_, 3 mg NaCl and 15 mg MoO_3_ powder were mixed together and placed in the middle of the tube furnace, and the temperature was maintained at 720 °C. For the growth of Sn*_x_*Mo_1−_ *_x_*S_2_ alloy, the two precursors were mixed together with the same ratio (15 mg MoO_3_ and 28 mg SnO_2_), and 5 mg NaCl was mixed together at the same time, and the temperature was kept at 710 °C. The same ratio of two growth precursors was to ensure that the Sn doping concentration of Sn*_x_*Mo_1−_ *_x_*S_2_ alloy was the same with that in the edge of Sn*_x_*Mo_1−_ *_x_*S_2_/MoS_2_ epitaxial heterostructures.

##### Working Electrode Preparation Method

Working electrode was made of Ti electrode on quartz glass by photolithography and evaporation technology. The CVD‐grown 2D materials were spin‐coated with PMMA and SiO_2_ was dissolved with NaOH. After being washed several times in water, the 2D transfer platform was used to transfer several samples to the Ti electrode. After baking, PMMA can be washed away with acetone and isopropanol.

##### Photoelectrochemical Measurements

All the measurements were carried out on an electrochemical workstation (CHI 750E, Chenhua, Shanghai) using a three‐electrode system, with Hg/HgCl_2_ reference electrode and a Pt mesh counter electrode. The electrolyte was 0.5 m Na_2_SO_4_ with pH ≈ 6.8. The linear‐sweep voltammogram curves, *I*–*t* curves, and chronoamperometry measurements were performed under AM 1.5G illumination (100 mW cm^−2^) from a class AAA solar simulator (XES‐40S3‐TT, San‐Ei Electric, Japan). The linear‐sweep voltammogram curves were performed by scanning the potential from negative to positive direction at a scan rate of 0.005 V s^−1^. EIS was obtained on the workstation at 1.23 V voltage under light irradiation, with the frequency ranging from 0.1 Hz to 100 kHz.

##### Characterization

The morphology of the samples was observed by optical microscopy (Nikon H600L). The SEM images were taken by Hitachi S‐4800 with 1–5 kV. The surface topography was examined with AFM (Bruker Dimension Icon AFM). The surface potential of these samples was quantitatively analyzed by KFPM (Bruker Dimension Icon AFM). Raman and PL spectra were performed using a WITec Alpha 300R spectrometer equipped with a 532 nm laser excitation and a CCD detector in a backscattering geometry. STEM images were acquired by using a JEOL 2100F microscope equipped with a cold field emission gun and double dodecaple correctors operated at 60 kV. The XPS was analyzed by the ESCALAB 250Xi XPS equipped with a monochromatic Al K*α* source (*λ* = 1486.6 eV). The XRD was performed by Bruker D8 ADVANCE. The field‐effect transistors (FETs) were fabricated with In/Au as metal contacts directly on SiO_2_/Si substrate without transferring by standard photolithography and thermal evaporation. The dynamics of photocarriers of these samples were monitored by the time‐resolved TAS. A regenerative amplifier system (Spitfire Ace, Spectra‐Physics, 800 nm wavelength, 120 fs pulse width, 250 Hz repetition rate) was used as the light source. The beam out of the amplifier was split into two beams: one was frequency doubled to get a beam of 400 nm as the pump beam; the other beam was focused into a sapphire plate to form the continuous white light with a range of 450–750 nm as the probe beam. The sub‐picosecond time delay between pump and probe beams was realized by a mechanic delay line (M‐ILS250CC, Newport, 250 mm). A spectrometer (AvaSpec‐2048L, Avantes) was placed behind the homemade microscope to get the transient signals.

## Conflict of Interest

The authors declare no conflict of interest.

## Supporting information

Supporting Information
