# Supplementary material for: Seamlessly Splicing Metallic SnxMo1− xS2 at MoS2 Edge for Enhanced Photoelectrocatalytic Performance in Microreactor
Source: Adv Sci (Weinh). 2020 Nov 16;7(24):2002172. doi: 10.1002/advs.202002172 (PMC7739950; doi:10.1002/advs.202002172)
Supplement: Supplementary file 1 — Supporting Information [file ADVS-7-2002172-s001.pdf]

© 2020 Wiley-VCH GmbH

ADVANCED SCIENCE
Open Access

Supporting Information

for *Adv. Sci.,* DOI: 10.1002/advs.202002172

Seamlessly Splicing Metallic $Sn_xMo_{1-x}S_2$ at MoS_2 Edge for Enhanced Photoelectrocatalytic Performance in Microreactor

Gonglei Shao, Yizhen Lu, Jinhua Hong, Xiong-Xiong Xue, Jinqiang Huang, Zheyuan Xu, Xiangchao Lu, Yuanyuan Jin, Xiao Liu, Huimin Li, Sheng Hu, Kazu Suenaga, Zheng Han, Ying Jiang, Shisheng Li, Yexin Feng, Anlian Pan, Yung-Chang Lin*, Yang Cao*, Song Liu**

Supporting Information

Seamlessly Splicing Metallic $Sn_xMo_{1-x}S_2$ at MoS_2 Edge for Enhanced Photoelectrocatalytic Performance in Microreactor

Gonglei Shao, Yizhen Lu, Jinhua Hong, Xiong-Xiong Xue, Jinqiang Huang, Zheyuan Xu, Xiangchao Lu, Yuanyuan Jin, Xiao Liu, Huimin Li, Sheng Hu, Kazu Suenaga, Zheng Han, Ying Jiang, Shisheng Li, Yexin Feng, Anlian Pan, Yung-Chang Lin*, Yang Cao*, Song Liu**

*Corresponding authors: anlian.pan@hnu.edu.cn; yclin.aist@gmail.com; yangcao@xmu.edu.cn; liusong@hnu.edu.cn

1. Density functional theory (DFT) calculations

First-principles calculations based on Density functional theory (DFT) were performed by the Vienna ab initio simulation package (VASP). The electronic correction interactions were described by the Perdew-Burke-Ernzerhof (PBE) functional with the generalized gradient approximation (GGA). The projector augmented wave (PAW) pseudo-potentials with cut-off energy of 500 eV were used. The 4×4 supercell was used to calculated electronic property of MoS_2 and $Sn_xMo_{1-x}S_2$. The Brillouin zone was sampled by 6×6×1 k-points within Monkhorst-Pack scheme. All atoms were optimized until the atomic forces were less than 0.02 eV/Å. A vacuum spacing of 15 Å was used to rule out negligible interlayer interaction.

2. The Fermi level and work function of MoS_2 and $Sn_xMo_{1-x}S_2$ calculated by DFT method

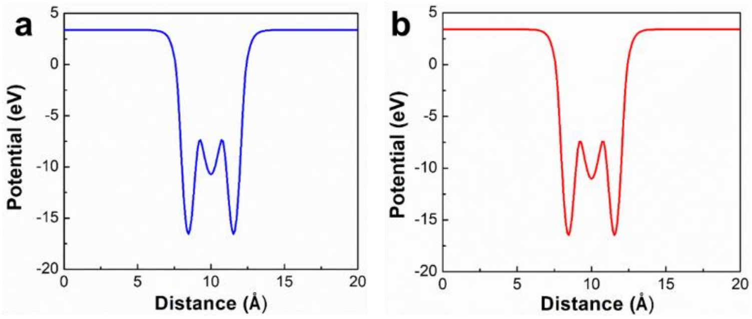

Figure S1. The calculated Fermi levels potential of (a) MoS_2 and (b) $Sn_xMo_{1-x}S_2$.

Table S1: The vacuum level, Fermi level and work function of MoS_2 and $Sn_xMo_{1-x}S_2$ calculated by DFT method

Name	Vacuum level (eV)	Fermi level (eV)	Work function (eV)
MoS_2	3.38	-2.12	5.50
$Sn_xMo_{1-x}S_2$	3.38	-2.11	5.49

3. The growth of MoS_2, $Sn_xMo_{1-x}S_2$ and epitaxial $Sn_xMo_{1-x}S_2/MoS_2$ heterostructures

We used the tube furnace for growing MoS_2, $Sn_xMo_{1-x}S_2$ and epitaxial $Sn_xMo_{1-x}S_2/MoS_2$ heterostructures under atmospheric pressure by chemical vapor deposition (CVD) in Supplementary Figure 2a. The atomic structure digrams of MoS_2, $Sn_xMo_{1-x}S_2$ and epitaxial $Sn_xMo_{1-x}S_2/MoS_2$ heterostructures were demonstrated in Supplementary Figure 2b. During the growth, SnO_2 and MoO_3 powders were selected as growth precursors in Supplementary Figure 2a, and the corresponding salt (NaCl) was added as catalyst and mixed with corresponding powders to lower the melting and boiling points. Conventional salt-assisted CVD method was used to grow 2D monolayer MoS_2,[1, 2] and the optical image of homogeneous MoS_2 was displayed in Supplementary Figure 5a. Two growth precursors (28 mg SnO_2 and 15 mg MoO_3) were evenly mixed to deposit ternary alloys on SiO_2/Si substrate to form $Sn_xMo_{1-x}S_2$ alloy, the optical image of $Sn_xMo_{1-x}S_2$ was shown in Supplementary Figure 5b. For growth mechanism of the alloy, the most important thing is the action mechanism of growing regional space. The SnO_2 powder and MoO_3 powder are first mixed together so that both together volatilize at the same temperature. Although the MoS_2 preferred to achieve the vapor pressure required for growth, for the mixed powders, the two kinds of SnO_2 powder and MoO_3 powder were completely mixed in the same growth space, so that only the $Sn_xMo_{1-x}S_2$ alloy could be grown on the substrate.

Among them, the two kinds of growth precursors with same ratio were spaced at an interval of 0.5 cm in the middle of the tube furnace as shown in Figure 2a and Supplementary Figure 2a, and prevented the volatile precursor from evaporating and depositing together on the SiO_2/Si substrate, resulting in the growth of $Sn_xMo_{1-x}S_2$ alloy. As for the separate precursor powder, due to the upstream MoO_3 reaches saturation vapor pressure first, the downstream volatile SnO_2 reaches saturation vapor pressure later. The two kinds of powders are separated at a certain space, the growth sequences are separated in space and time. Thus, such spatial spacing results in that MoS_2 is already prefered to grow in the central encounter, while SnS_2

diffuses upstream later and mixed with MoS_2, leads to grow $Sn_xMo_{1-x}S_2$ at edge of MoS_2 for $Sn_xMo_{1-x}S_2/MoS_2$ heterostructure. Interestingly, two different color modules were spliced together to form a concentric heterogeneous structure with distinct colors, which emerged from the optical image in Supplementary Figure 3 and Supplementary Figure 5c.

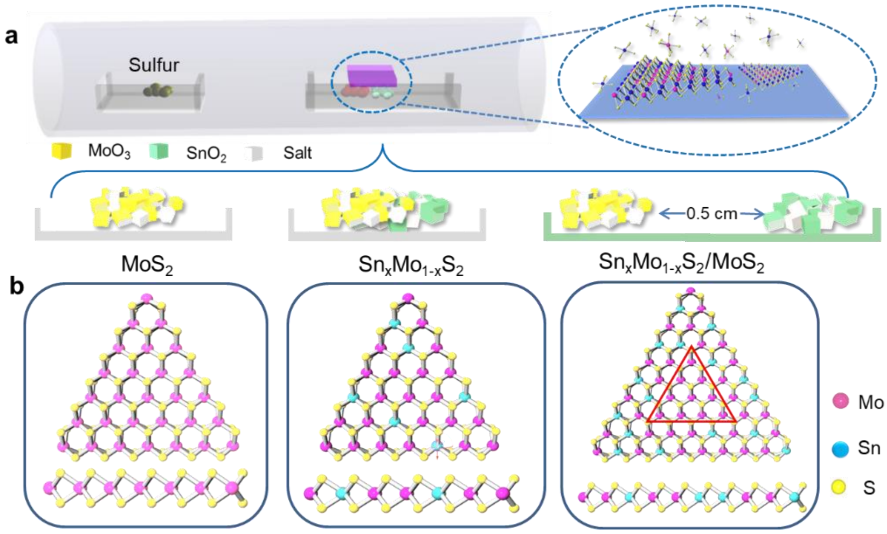

Figure S2. The growth schematic diagram of MoS_2, $Sn_xMo_{1-x}S_2$ and $Sn_xMo_{1-x}S_2/MoS_2$ heterostructures. a) Growth schematic diagrams of three kinds of 2D materials by adjusting precursors. b) Atomic structure diagrams of three kinds of 2D materials.

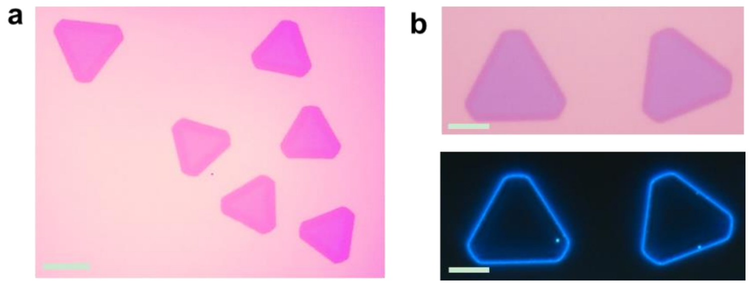

Figure S3. a) Optical image of $Sn_xMo_{1-x}S_2/MoS_2$ heterostructures. Scale bar: 20 μm. b) Optical images under bright-field (upper) and dark-field optical microscopes (bottom). Scale bar: 10 μm.

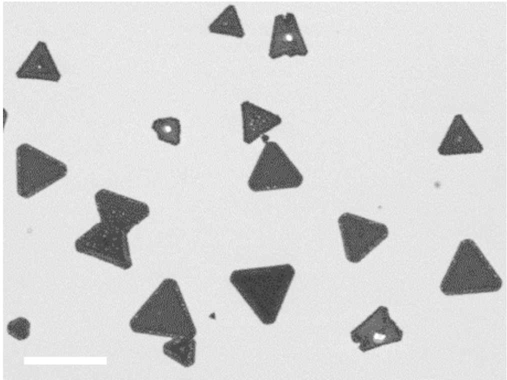

Figure S4. The SEM image of epitaxial $Sn_xMo_{1-x}S_2/MoS_2$ heterostructures. Scale bar: 20 µm.

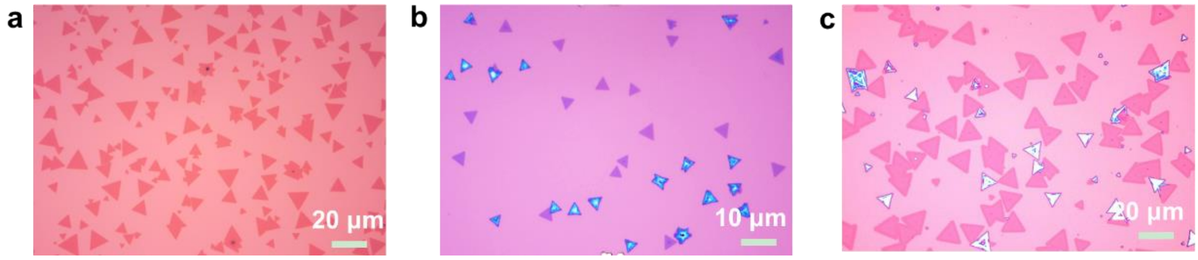

Figure S5. The optical images of a) MoS_2, b) $Sn_xMo_{1-x}S_2$ alloy and c) epitaxial $Sn_xMo_{1-x}S_2/MoS_2$ heterostructures. Scale bar: 20 µm.

4. Basic characterization of MoS_2

The thickness of monolayer MoS_2 was analyzed by atomic force microscope (AFM), the monolayer properties were revealed, with a thickness of 0.76 nm (Supplementary Figure 6a). At the same time, 532 laser was used to perform Raman and PL test, Raman spectrum showed two typical characteristic peaks, the E^1_{2g} peak at 385.4 cm^{-1} and the A_{1g} at 405.9 cm^{-1} in Supplementary Figure 6d.[2, 3] The obvious band gap near 1.82 eV (Supplementary Figure 6e) illustrated that the single-layer MoS_2 showed distinct direct band gap.[1] At the same time, Raman mapping at 405 cm^{-1} (Supplementary Figure 6b) and PL mapping at 1.82 eV (Supplementary Figure 6c) showed the color of the uniform distribution. These results illustrated the MoS_2 possessed good crystallinity and single crystal features.

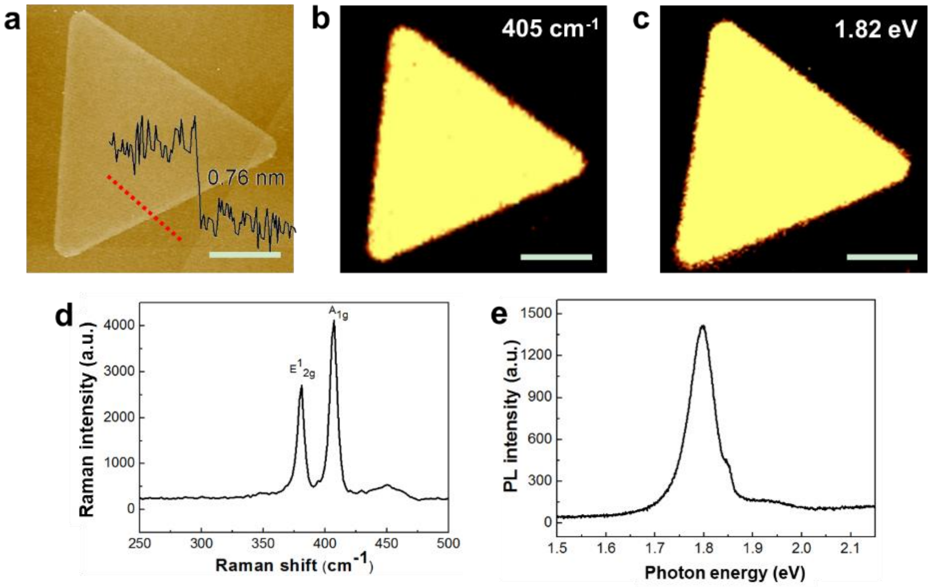

Figure S6. a) AFM image, b) Raman mapping, c) PL mapping, d) Raman and e) PL spectrum of MoS_2. Scale bar: 5 μm.

5. Basic characterization of $Sn_xMo_{1-x}S_2$ alloy

The thickness of monolayer $Sn_xMo_{1-x}S_2$ was analyzed by AFM, the monolayer property was revealed, with a thickness of 0.87 nm (Supplementary Figure 7a). The $Sn_xMo_{1-x}S_2$ alloy showed obviously different properties with pure MoS_2 in Raman and PL. Apart from E^1_{2g} and A_{1g} peaks of Raman, three new vibration peaks (J_1, J_2 and J_3) appeared at 182.9, 220.4 and 346.1 cm^{-1} (Supplementary Figure 8a). This result may be due to the distortion of the lattice caused by doping Sn, resulted in additional vibration patterns.[3, 4] At the same time, PL spectrum of the sample was collected (Supplementary Figure 8b), without any peak. The absence of PL excitation behavior further indicated that the $Sn_xMo_{1-x}S_2$ alloy became a metallic 2D material. The apparent phenomenon was caused by the doped Sn, resulted in the change of the band structure, which induced the quenching of PL. The result was basically consistent with reports.[4-6]

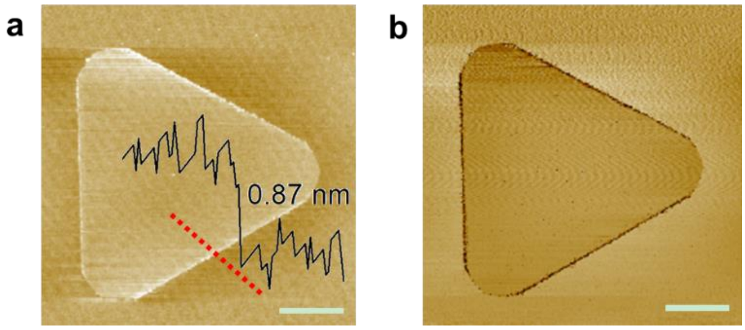

Figure S7. a) AFM image of $Sn_xMo_{1-x}S_2$ alloy. b) Corresponding phase diagram of AFM image. Scale bar: 5 μm.

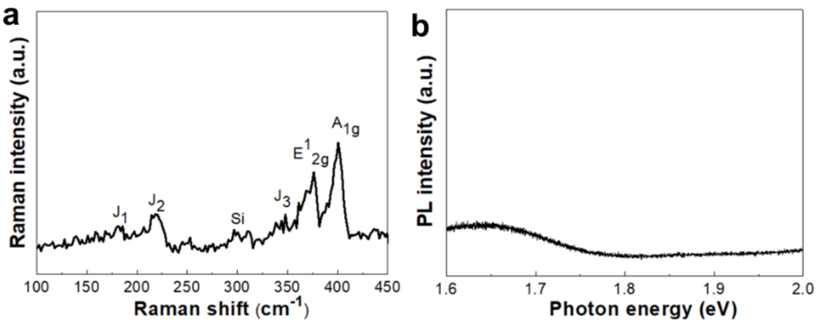

Figure S8. a) Raman spectrum and b) PL spectrum of $Sn_xMo_{1-x}S_2$ alloy.

6. Electrical behavior of MoS_2 and $Sn_xMo_{1-x}S_2$ alloy

The lithography and metal evaporation methods were used to prepare the field effect transistors (FETs) of MoS_2 and $Sn_xMo_{1-x}S_2$ alloy. The metallic conductor in nature of $Sn_xMo_{1-x}S_2$ alloy was also demonstrated by electrical behavior test. Monolayer MoS_2 demonstrated obvious n-type semiconductor electrical transmission behavior (Supplementary Figure 9). While electrical transmission behavior of $Sn_xMo_{1-x}S_2$ alloy was not regulated by V_G, I_{DS} increased linearly with the increase of V_{DS} (Supplementary Figure 10). It further illustrated that the Sn-doping led MoS_2 transit from semiconductor to metal, and $Sn_xMo_{1-x}S_2$ alloy clearly displayed metal electrical behavior. The I_{DS}-V_{DS} curve further demonstrated that metallic $Sn_xMo_{1-x}S_2$ alloys exhibited good Ohmic contact with electrodes (Supplementary Figure 10c). These results of metallic $Sn_xMo_{1-x}S_2$ electrical properties also provide direct evidence for the improved electron transmission and lowered contact resistance.

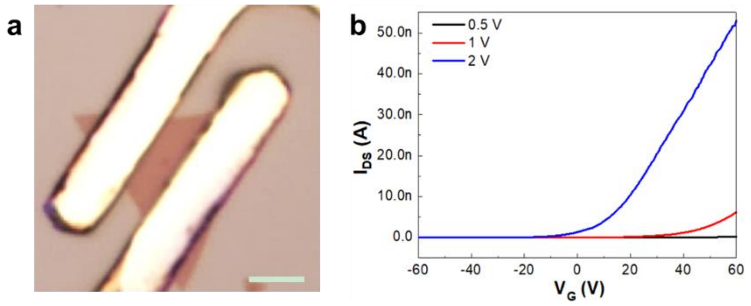

Figure S9. a) The optical image of MoS_2 device. Scale bar: 5 μm. b) Source-drain current (I_{DS}) versus gate voltage (V_G) characteristics of pure MoS_2.

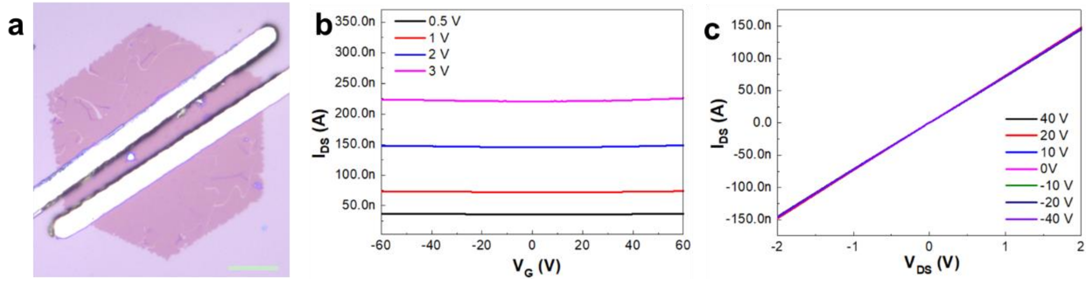

Figure S10. a) The optical image of $Sn_xMo_{1-x}S_2$ alloy device. Scale bar: 5 μm. b) Source-drain current (I_{DS}) versus gate voltage (V_G) characteristics of $Sn_xMo_{1-x}S_2$ alloy. c) The Output curves (I_{DS}-V_{DS}) of $Sn_xMo_{1-x}S_2$ alloy.

7. Growth process prediction of epitaxial $Sn_xMo_{1-x}S_2/MoS_2$ heterostructures

The lateral M-S heterostructures were created in the sequence indicated in Figure 2c, under the conditions of similar growth temperature of the two kinds of 2D materials (MoS_2 and SnS_2), and different growth vapor pressure of the two materials at the same temperature. The conversion from one component to two components permitted the epitaxial growth of $Sn_xMo_{1-x}S_2$ directly on existing MoS_2 crystal edges. The change in conditions can be explained by the difference in volatility of the source powders combined with atomic compatibility. This result can be clearly confirmed from the thermo gravimetric energy spectrum of SnS_2 and MoS_2 (Supplementary Figure 11), MoS_2 firstly decomposed at about 400 °C, while SnS_2 decomposed at about 600 °C. It showed that Mo source was volatilized preferentially to Sn source and reached saturated vapor pressure, providing direct support for the *in-situ* growth of this heterostructures in turn. Interestingly, the reproducibility of our results suggested that a more sophisticated set-up with independent control of the vapor components could be developed if necessary to create programmable heterostructures for complex device applications.[7]

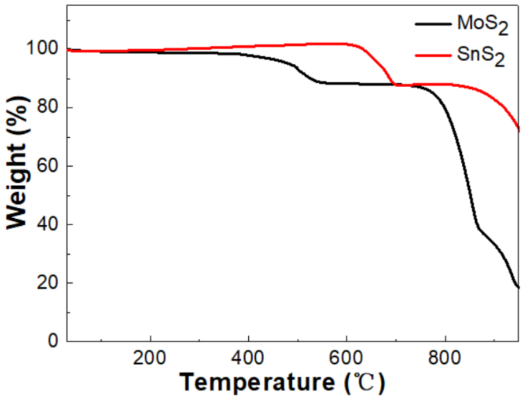

Figure S11. The thermogravimetric energy spectrum analysis of SnS_2 and MoS_2.

8. X-ray diffraction (XRD) analysis of epitaxial $Sn_xMo_{1-x}S_2/MoS_2$ heterostructures

XRD was further used to characterize the composition of these composite metal atomic vapor deposited on SiO_2/Si substrate (Supplementary Figure 12). Two unusually prominent peaks appear at 14.4^o and 15.0^o, corresponding to the crystal (002) plane peak of MoS_2,[8] and the (001) plane of SnS_2,[9] respectively. Simultaneously, the unusually distinct peaks relative to other crystal surfaces indicated that these materials were tiled on the substrate in the form of 2D plane, illustrating the 2D properties of these structures. The presence of SnS_2 was due to the deposition of some SnS_2 crystal on the substrate in the later growth process (except for the growth of $Sn_xMo_{1-x}S_2$ alloys). These results were also proved from the side that metal component of the vapor above the substrate was predominantly Mo and Sn.

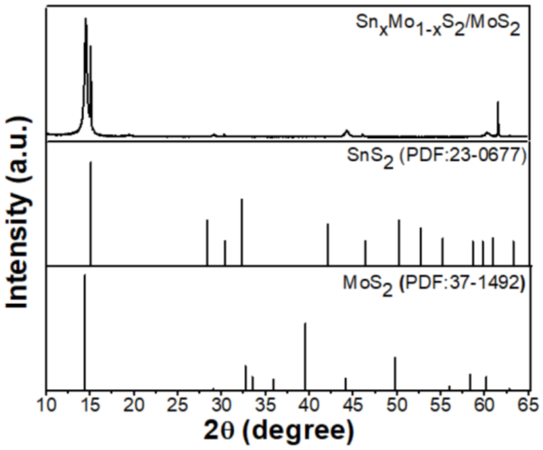

Figure S12. The XRD data of the $Sn_xMo_{1-x}S_2/MoS_2$ heterostructures deposited on SiO_2/Si substrate.

9. The KPFM test of MoS_2, $Sn_xMo_{1-x}S_2$ and epitaxial $Sn_xMo_{1-x}S_2/MoS_2$ heterostructures

KPFM devices were prepared with Au/Cr electrodes by using electron beam lithography and thermal evaporation, and then connected the 2D material samples with AFM machine through the electrode, and the Peak Force KPFM-AM mode of Bruker Dimension Icon AFM was applied to test the surface potential difference. The optical picture of the triangular 2D MoS_2, $Sn_xMo_{1-x}S_2$ and epitaxial $Sn_xMo_{1-x}S_2/MoS_2$ heterostructures connected electrode was displayed in Supplementary Figure 13a, 13d and 13g, respectively. The pure MoS_2 and metallic $Sn_xMo_{1-x}S_2$ alloy exhibited the properties of single layer by AFM test, without apparent surface potential difference at the interface (Supplementary Figure 13c and 13f). However, the surface potential of the alloy was significantly higher than that of MoS_2 by contrast brightness. Interestingly, a clear surface potential interface in epitaxial $Sn_xMo_{1-x}S_2/MoS_2$ heterostructure was observed (Supplementary Figure 13i), the brightness of the outside was significantly higher than that of the inside. This phenomenon conformed to the test results of individual MoS_2 and metallic $Sn_xMo_{1-x}S_2$, indicating different Fermi-level between semiconducting MoS_2 and metallic $Sn_xMo_{1-x}S_2$. The work function of metallic $Sn_xMo_{1-x}S_2$ was distinctly smaller than that of MoS_2 relative to the vacuum level.

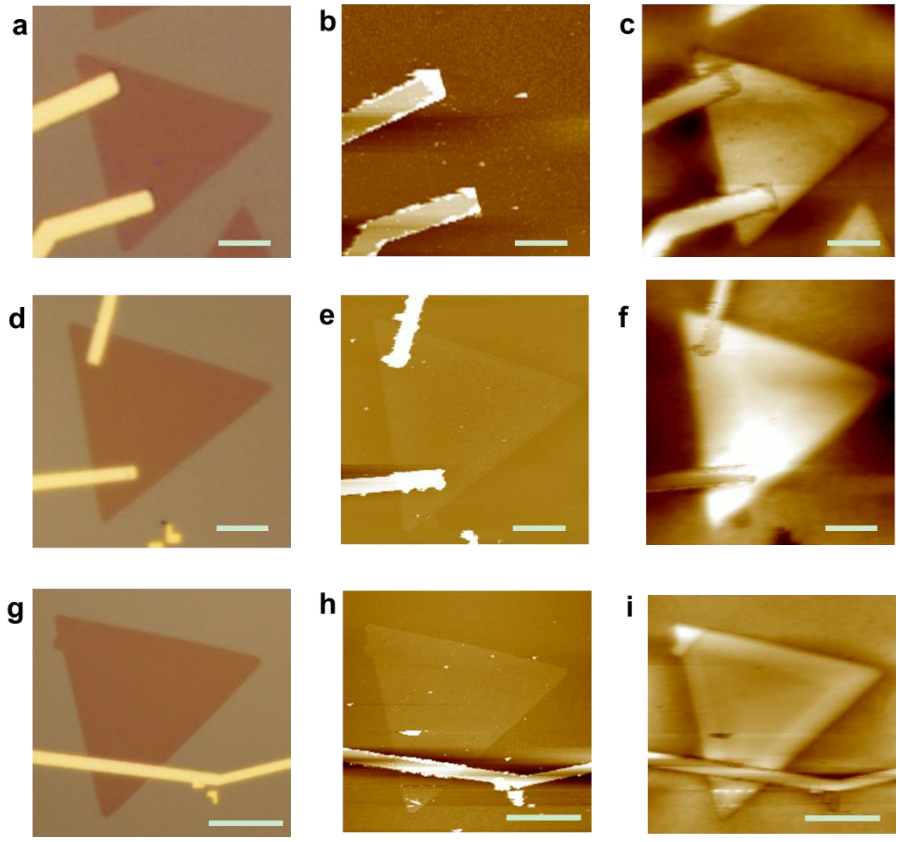

Figure S13. a-c) The optical images, AFM images and KFPM images of MoS_2, d-f) $Sn_xMo_{1-x}S_2$ alloy and g-i) epitaxial $Sn_xMo_{1-x}S_2/MoS_2$ heterostructures. Scale bar: 10 μm.

10. The stability test of epitaxial $Sn_xMo_{1-x}S_2/MoS_2$ heterostructures

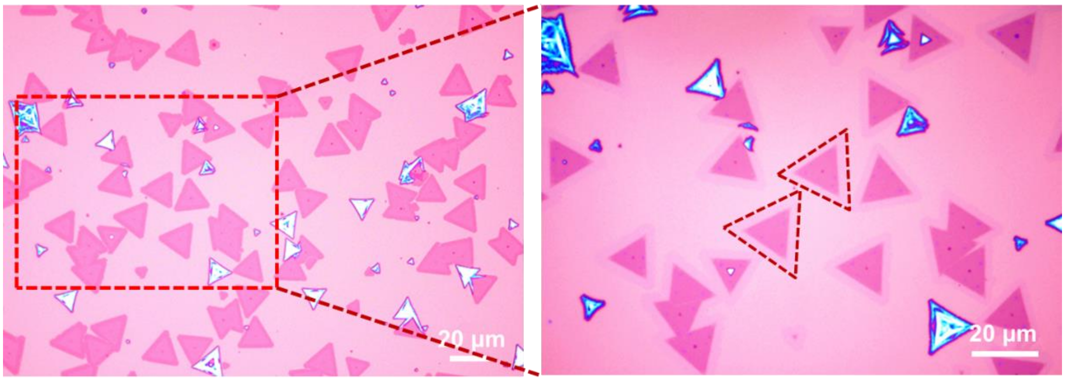

Figure S14. The optical images of stability test of epitaxial $Sn_xMo_{1-x}S_2/MoS_2$ heterostructures after 2 weeks.

11. X-ray photoelectron spectroscopy (XPS) analysis of epitaxial $Sn_xMo_{1-x}S_2/MoS_2$ heterostructures

The sample of M-S $Sn_xMo_{1-x}S_2/MoS_2$ epitaxial heterostructures was monitored by XPS characterization (Supplementary Figure 15). Firstly, the diffraction peaks of Mo 3d were detected, which were distributed at the locations of 232.2 eV and 229.3 eV (Supplementary Figure 15a). Meanwhile, two binding energies of S located at 163.2 eV and 161.9 eV were respectively corresponding to S $2p_{1/2}$and S $2p_{3/2}$ (Supplementary Figure 15b), which can be peak divided into the binding energy of S-Sn (163.4 eV and 162.1 eV) and S-Mo (163.1 eV and 161.8 eV). Moreover, the binding energy difference with S-Sn and S-Mo was 0.3 eV. For the Sn 3d spectrum in Supplementary Figure 15c, the splitting of these peaks with Sn $3d_{3/2}$ and Sn $3d_{5/2}$ indicated that Sn had a variety of binding states. The double peaks belonged to Sn binding energy in SnS_2 (494.9 eV and 486.1 eV),[9, 10] which was 0.9 eV lower than that of $Sn_xMo_{1-x}S_2$ (495.8 eV and 487.0 eV). This indicated that part of the Sn binding energy existed in $Sn_xMo_{1-x}S_2$, and part of the Sn binding energy existed in SnS_2. While there was Sn binding energy in SnS_2 because the whole substrate of the deposition sample was irradiated in the surface area by XPS. These results further validated the presence of Sn doping in $Sn_xMo_{1-x}S_2$ alloy through S-Sn bond analysis.

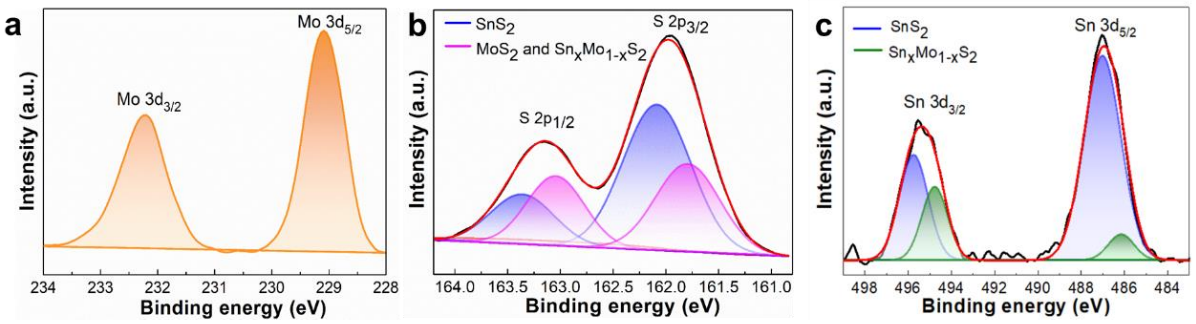

Figure S15. XPS analysis at Mo 3d spectra a) and S 2p spectra b) and Sn 3d spectra c) of $Sn_xMo_{1-x}S_2/MoS_2$ heterostructures.

12. The photoelectrocatalytic performance test in microreactor

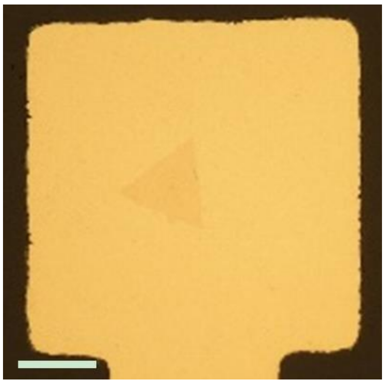

Figure S16. The optical image of epitaxial $Sn_xMo_{1-x}S_2/MoS_2$ heterostructures transferred to the quartz glass with Ti metal electrode for OER test. Scale bar: 20 μm.

To verify this charge transport mechanism at interface, electrochemical impedance spectroscopy (EIS) tests were also examined. The interface reaction dynamics of electrode was evaluated by the EIS (Supplementary Figure 17) and the inset was equivalent circuit to fit the plots. The charge-transfer resistances (Rct) at the photoanode/electrolyte interface were summarized in Supplementary Table 2, in which, Rct of $Sn_xMo_{1-x}S_2/MoS_2$ (16.86 Ω) was dramatically reduced with six orders of magnitude compared to that of MoS_2 ($3.6*10^6$ Ω). $Sn_xMo_{1-x}S_2/MoS_2$ and $Sn_xMo_{1-x}S_2$ alloy revealed similar Rct due to rapid charge transfer with lower resistance. Hence, the formed M-S heterojunction with splicing metallic $Sn_xMo_{1-x}S_2$ promoted the fast charge transfer across the heterostructure-electrolyte interface and led to improved photoelectric catalysis performance.

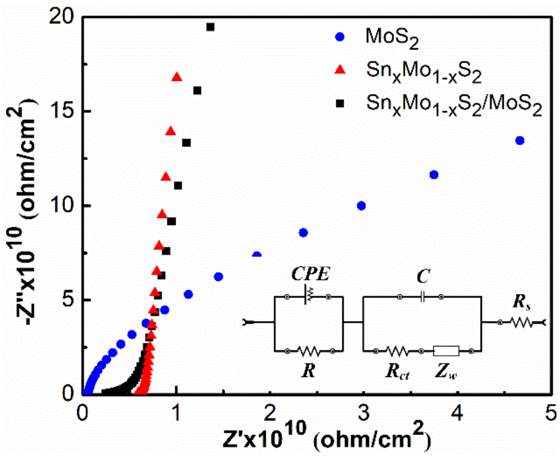

Figure S17. EIS-Nyquist plots of photoanodes under illumination. The inset showed the equivalent circuit to fit the plots. The equivalent circuit parameters included: the solution resistance (Rs), Warburg impedance (Zw), charge transfer resistance (R and Rct) meaned the impedance at the interface between Ti/photoanode and the photoanode/electrolyte, respectively. C was the capacitance of electrode–electrolyte interface and CPE was the mass capacitance.

Table S2. The charge-transfer resistance (Rct) at the photoanode/electrolyte interface of MoS_2, $Sn_xMo_{1-x}S_2$ alloy and epitaxial $Sn_xMo_{1-x}S_2/MoS_2$ heterostructures.

Samples	Rct
$Sn_xMo_{1-x}S_2/MoS_2$	16.86 Ω
MoS_2	$3.6*10^6$ Ω
$Sn_xMo_{1-x}S_2$	0.01 Ω

13. UV/Vis absorption spectra and band-gap spectra of MoS_2 and $Sn_xMo_{1-x}S_2$.

The UV/Vis absorption was detected with UV-visible absorption spectrum for measuring the MoS_2 light-harvesting capabilities in Supplementary Figure 19, exciton absorption peaks

caused by direct band gap transitions were 625 and 650 nm for MoS_2, the peak near 450 nm was due to the optical band gap transition between the conduction and valence bands. We can use the Tauc equation to get the direct band gap value (the intersection of the black line and the abscissa) according to the UV-Vis spectrum (Supplementary Figure 19a), and the direct band gap value of MoS_2 was 1.85eV (Supplementary Figure 19b).

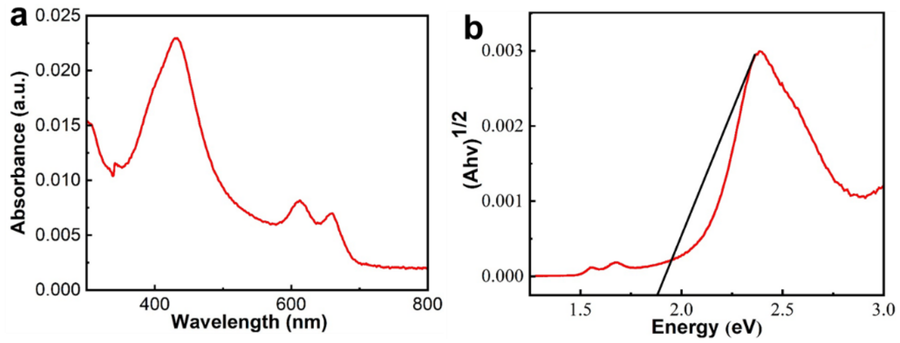

Figure S18. a) UV-Vis spectra of MoS_2. b) UV-Vis spectra of MoS_2 in energy band.

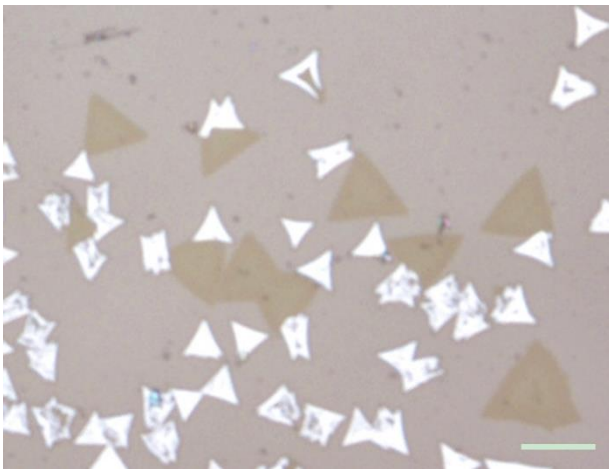

Figure S19. The optical image of epitaxial $Sn_xMo_{1-x}S_2/MoS_2$ heterostructures transferred to the mica substrate for TAS test. Scale bar: 20 µm.

References

[1] S. Wang, Y. Rong, Y. Fan, M. Pacios, H. Bhaskaran, K. He, J. H. Warner, *Chem. Mater.* 2014, **26**, 6371.

[2] Q. Fu, X. Wang, J. Zhou, J. Xia, Q. Zeng, D. Lv, C. Zhu, X. Wang, Y. Shen, X. Li, Y. Hua, F. Liu, Z. Shen, C. Jin, Z. Liu, *Chem. Mater.* 2018, **30**, 4001.

[3] L. Liu, J. Wu, L. Wu, M. Ye, X. Liu, Q. Wang, S. Hou, P. Lu, L. Sun, J. Zheng, *Nat. Mater.* 2018, **17**, 1108.

[4] J. Zhu, Z. Wang, H. Yu, N. Li, J. Zhang, J. Meng, M. Liao, J. Zhao, X. Lu, L. Du, *J. Am. Chem. Soc.* 2017, **139**, 10216.

[5] S. Mignuzzi, A. J. Pollard, N. Bonini, B. Brennan, I. S. Gilmore, M. A. Pimenta, D. Richards, D. Roy, *Phys. Rev. B* 2015, **91**, 195411.

[6] K. Bogaert, S. Liu, T. Liu, N. Guo, C. Zhang, S. Gradecak, S. Garaj, *Sci. Rep.* 2018, **8**, 12889.

[7] C. Huang, S. Wu, A. M. Sanchez, J. J. P. Peters, R. Beanland, J. S. Ross, P. Rivera, W. Yao, D. H. Cobden, X. Xu, *Nat. Mater.* 2014, **13**, 1096.

[8] Y. Yu, G. H. Nam, Q. He, X. J. Wu, K. Zhang, Z. Yang, J. Chen, Q. Ma, M. Zhao, Z. Liu, F. R. Ran, X. Wang, H. Li, X. Huang, B. Li, Q. Xiong, Q. Zhang, Z. Liu, L. Gu, Y. Du, W. Huang, H. Zhang, *Nat. Chem.* 2018, **10**, 638.

[9] G. Shao, X. X. Xue, X. Zhou, J. Xu, Y. Jin, S. Qi, N. Liu, H. Duan, S. Wang, S. Li, M. Ouzounian, T. S. Hu, J. Luo, S. Liu, Y. Feng, *ACS nano* 2019, **13**, 8265.

[10] G. Shao, X. X. Xue, B. Wu, Y. C. Lin, M. Ouzounian, T. S. Hu, Y. Xu, X. Liu, S. Li, K. Suenaga, Y. Feng, S. Liu, *Adv. Funct. Mater.* 2020, **30**, 1906069.
